# Routing Selection Algorithm for Mobile Ad Hoc Networks Based on Neighbor Node Density

**DOI:** 10.3390/s24020325

**Published:** 2024-01-05

**Authors:** Xiaolin Li, Xin Bian, Mingqi Li

**Affiliations:** 1 Shanghai Advanced Research Institute, Chinese Academy of Sciences, Shanghai 201210, China; lixiaolin@sari.ac.cn (X.L.); bianx@sari.ac.cn (X.B.); 2 University of Chinese Academy of Sciences, Beijing 100049, China

**Keywords:** AODV, ND-AODV, CND-AODV, ad hoc, neighbor nodes, route discovery

## Abstract

In the process of data transmission in mobile ad hoc networks, it is essential to establish optimal routes from source nodes to destination nodes. However, as network density increases, this process is often accompanied by a significant rise in network overhead. To address this issue, the ND-AODV (neighborhood density AODV) protocol has been introduced, which reduces the probability of transmitting control information in high-density node environments to mitigate network overhead. Nevertheless, this may come at the cost of reduced routing accuracy, potentially leading to unnecessary resource wastage in certain scenarios. Furthermore, ND-AODV does not comprehensively consider the location of the receiving nodes, which limits its ability to reduce network overhead effectively. To overcome these limitations, this paper introduces a novel routing approach, known as CND-AODV (common neighborhood density AODV). In comparison to ND-AODV, CND-AODV offers a more comprehensive solution to the challenges posed by high-density network environments. It intelligently processes control information based on the special positioning of the receiving nodes, thereby significantly reducing unnecessary network overhead. Through simulation experiments comparing performance metrics such as throughput, packet delivery rate, and latency, the results clearly indicate that CND-AODV substantially decreases network overhead, enhancing network performance. Compared to ND-AODV, this innovative routing approach exhibits significant advantages. It provides a more efficient and reliable solution for ad hoc networks in high-density environments.

## 1. Introduction

### 1.1. Background

With the rapid development of society, there is an increasing demand for wireless connectivity, and the required quality is becoming higher [1,2]. Generally, wireless networks can be categorized into two types: infrastructure-based networks, which are typically controlled by a central network such as GSM (Global System for Mobile Communications), IoT (internet of things) [3], or Wi-Fi, and infrastructureless networks, which consist of a network of interconnected devices without a central controlling node, e.g., ad hoc networks.

Mobile ad hoc networks [4] fall under the category of ad hoc networks and represent a type of wireless network. Comprising a set of mobile nodes, they have the capability to autonomously establish a network without the need for preconfigured infrastructure. Furthermore, they can create instantaneous communication networks without an internet connection. These nodes interconnect through self-organization and distributed methods to facilitate the exchange of information and data.

Mobile ad hoc networks were initially developed for military applications, but they are now widely used in various fields such as emergency medical response, unmanned aerial vehicles (UAVs), and self-organizing sensor networks. Mobile ad hoc networks have several advantages. 1. Damage resistance: As a decentralized network, mobile ad hoc networks do not rely on single nodes. This means that even if one or more nodes fail or are destroyed, the network can still function properly. This network topology also provides the capability to rapidly adapt to the addition or removal of nodes. 2. Flexibility and Scalability: Mobile ad hoc networks are versatile and can adapt to various types of network environments. These advantages make mobile ad hoc networks very useful in scenarios that require fast deployment, high flexibility, and fast response [5,6].

However, there are still some challenges and limitations in mobile ad hoc networks. For instance, in scenarios where nodes are moving rapidly, the quick changes in network topology can lead to the failure and recalibration of routing paths. This can result in the failure and recalculation of routing paths, causing some nodes to send a large number of broadcast messages to other nodes within a short period. These messages overlap with the regular messages being transmitted by nodes, resulting in momentary traffic surges and congestion that exceed the network’s bandwidth and resource capacity. As a consequence, network performance deteriorates, and in severe cases, it might lead to complete network interruption. This phenomenon is known as a network storm. To address this issue, researchers have developed various strategies and technologies to optimize the performance of mobile ad hoc networks and reduce the risk of network storms. Among these approaches, the most commonly used methods include congestion control and routing protocols, such as AODV (ad hoc on-demand distance vector). These technologies and protocols help reduce unnecessary broadcast messages and optimize routing paths, thereby preventing the occurrence of network storms.

With the evolving demands, these methods perform admirably in scenarios with a relatively smaller number of mobile devices. However, as the number of mobile devices increases, network performance often experiences a significant decline. This is because, with the growing number of devices, communication activities within the network become more complex and intensive, leading to issues like network congestion and performance degradation. In ND-AODV, the introduction of neighbor node density serves as a solution. As the number of neighboring nodes increases, the probability of nodes forwarding control information gradually decreases, thereby effectively alleviating network congestion. Nevertheless, this approach does not adequately account for the precise location of the receiving nodes, potentially resulting in unnecessary wastage of network resources. Therefore, there is a need for more intelligent and efficient methods to cope with the ever-expanding count of mobile devices, with the aim of enhancing network performance and reducing resource wastage.

In consideration of the aforementioned issues, this study introduces an innovative optimization algorithm based on common node density, known as CND-AODV. This algorithm not only effectively addresses the challenges posed by a high neighbor node density, but also takes into account the location of the receiving nodes, thus further reducing wastage of network resources and enhancing network performance.

### 1.2. Related Work

In the field of mobile ad hoc networks, numerous experts are dedicated to researching how to address the issue of network storms caused by the broadcast of control messages. This section primarily focuses on previous work in this area, exploring how different environmental factors have been utilized to tackle the problem, as well as the remaining challenges.

In [7], an optimized protocol called ND-AODV is proposed, which primarily considers utilizing the number of neighbor nodes to determine whether a node exists in a high-density environment. If it is determined that the node is in a high-density environment, the protocol uses a ratio between a threshold and the number of neighbor nodes to calculate its forwarding probability. This algorithm aims to reduce network overhead, but it does encounter some problems. One of the problems is the lack of precise information about the specific positions of neighbor nodes. This can lead to potential route loss if the node happens to be on a critical route towards the destination node.

In [8], a routing approach called PAODV (probability AODV) is proposed, which also takes into account the node density. The idea behind PAODV is that as the network density increases and each node has more neighbors, the probability of finding multiple routes from the source to the destination also increases. Therefore, limiting the number of RREQ (route request) packets that are rebroadcast by intermediate nodes can reduce the overall network overhead. If the node density is sparse, a higher forwarding probability (p1) is used for forwarding. Conversely, if the node density is high, a lower forwarding probability (p2) is used for controlling the forwarding of control messages. In [9], an analysis is provided to determine the optimal values of forwarding probabilities for both high-density and low-density scenarios. However, these forwarding probabilities are set before running the simulations and do not consider the specific network dynamics. Therefore, in situations where the node density is either too low or too high, there may still be issues of routes not being discovered or excessive network overhead, and there is not good scalability.

In [10,11], a routing metric optimization method called AODV-ETX (AODV with expected transmission count) is proposed. It selects routes by estimating the optimal route to neighbor nodes based on the expected transmission count (ETX) rather than the hop count metric used in the original AODV. By optimizing the routing metric, the goal is to reduce network overhead. In this method, ETX is primarily based on the reciprocal of the success probability of new control information called link probe packets (LPP) exchanged between nodes. The optimal ETX value from the source to the destination is determined by the best new metric obtained from the nodes along the route. However, this method introduces new control information (LPP), which can lead to increased control message overhead in networks with rapidly changing topologies. As a result, AODV-ETX is more suitable for mobile ad hoc networks with relatively stable topologies. In [12], an optimization method for ETX is proposed, consisting of three variants: L-ETX (light ETX), LR-ETX (light reverse ETX), and PLR-ETX (power light reverse ETX). These three methods also optimize the routing metric but do not specifically address the issue of rapidly changing topologies.

In [13], a self-adaptive multipath routing algorithm called GA-AOMDV (genetic algorithm AOMDV) is introduced. This algorithm selects nodes with high remaining energy as routers, reducing energy consumption during the data packet communication process. In [14], another adaptive algorithm called RLLMR (reliable low-latency multipath routing) is proposed. It dynamically adjusts the parallel routing multipath algorithm based on service requirements to meet different services’ latency demands while conserving routing resources. Moreover, it introduces a local route repair scheme based on maintenance nodes to enhance the reliability of the communication process. Both of these adaptive algorithms seek the best routes based on different factors but do not consider the issue of excessive network resource consumption that may occur in densely populated nodes.

In [15], a novel routing metric called Enhanced-Ant-AODV is proposed. It utilizes a combination of parameters such as node density, signal strength, distance, and remaining node energy as the routing metrics. The ant colony optimization algorithm is employed to find an optimal route based on these parameters. To ensure the selection of an optimal route that avoids nodes with low energy levels and significant signal strength differences, the routing decision process avoids forwarding routing control information through such nodes. This helps reduce network overhead to some extent. The consideration of multiple factors in the routing metric allows it to adapt well to different environments and find routes that effectively meet the transmission requirements. However, in environments with high node density, this approach may still experience significant network overhead. Further research is needed to address this limitation and develop more efficient strategies for routing control in dense node environments.

The remainder of this paper is organized as follows: Section 3 reviews the cyber storm problem and related solutions. The following sections outline the key points covered. In Section 4, the detailed process and the existing problems of ND-AODV are introduced, and then a new scheme CND-AODV is proposed. Section 5 presents a simulation experiment on the NS3 platform to evaluate the performance of the proposed CND-AODV. Finally, Section 6 summarizes the whole paper and gives the direction of future work.

## 2. Notation

This section serves as a comprehensive introduction to the parameter variables used across the entire document. These parameters are prominently consolidated and detailed in Table 1. Moreover, their significance and utilization are elaborated upon in the subsequent expressions presented throughout the text.

## 3. Node-Density-Based Routing Method

### 3.1. AODV

Ad hoc network protocols are protocols used to manage and control ad hoc networks, which are composed of many distributed nodes that can automatically discover and establish connections. These protocols facilitate data transmission and routing within the network. Depending on the discovery strategy, ad hoc network protocols can be categorized into proactive routing protocols, such as destination-sequenced distance vector (DSDV) [16] and optimized link state routing (OLSR) [17], and reactive (on-demand) routing protocols, such as ad hoc on-demand distance vector (AODV) [18,19] and dynamic source routing (DSR) [20].

Due to the large amount of information that needs to be maintained in the node routing table, proactive routing protocols are not suitable for highly dynamic mobile ad hoc networks with frequent topology changes. Therefore, in this paper, the optimization of mobile ad hoc networks is based on the reactive (on-demand) routing protocol AODV. AODV can be seen as a combination of the DSR and DSDV protocols and consists of two main processes: route discovery and route maintenance. The entire routing process is primarily determined by the routing table, route request (RREQ), route response (RREP), route error (RERR), and HELLO messages.

**Figure 1:**
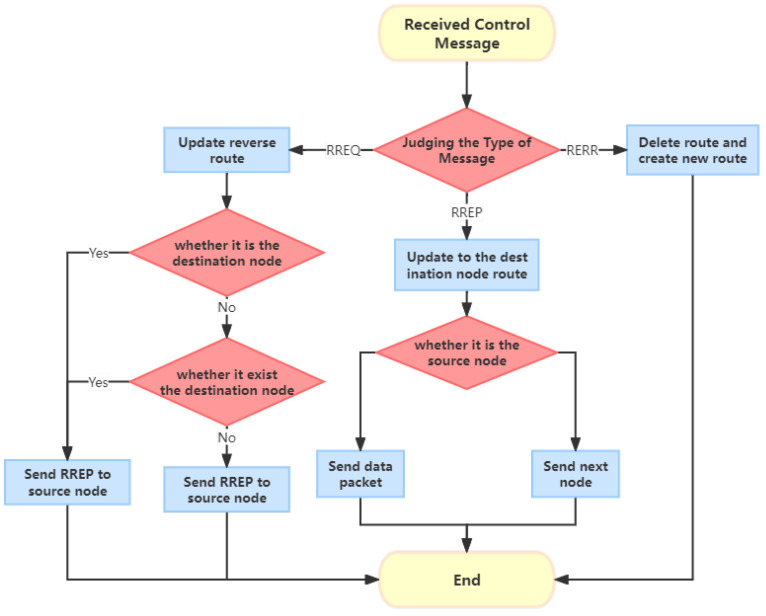
The route discovery procedure of the AODV protocol.

Route discovery: The source node initiates the route discovery process by broadcasting a route request (RREQ) message to its neighbor nodes. Each neighbor node checks its own routing table to determine if it has a route to the destination node. If it does, it unicasts a route response (RREP) message back to the source node, following the original path. If the neighbor node does not have a route to the destination, it continues broadcasting the RREQ message to its own neighbor nodes, and the process continues until the destination node is discovered. This process is illustrated in Figure 1.

**Figure 2:**
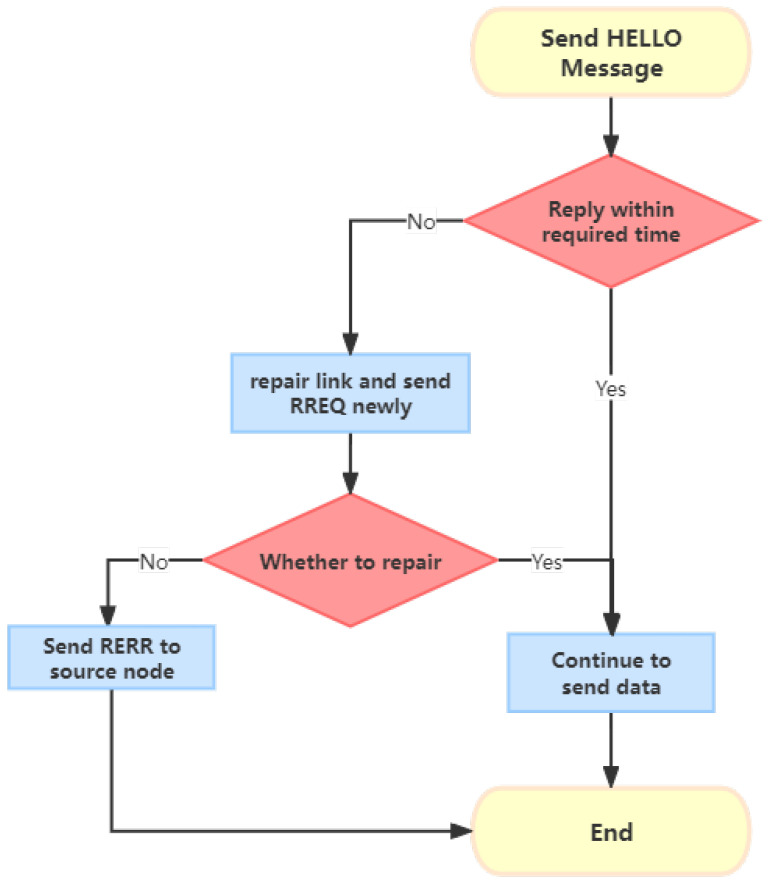
The route maintenance of the AODV protocol.

Route maintenance: During the data transmission phase, nodes periodically send HELLO control messages to their neighbor nodes to maintain the routing table information. If a node becomes unreachable, it sends a route error (RERR) message to the source node to initiate route repair. If a node detects a change in the network, it sends the information to the appropriate node to update the routing information and maintain the connectivity of the network. This process is depicted in Figure 2.

**Figure 3:**
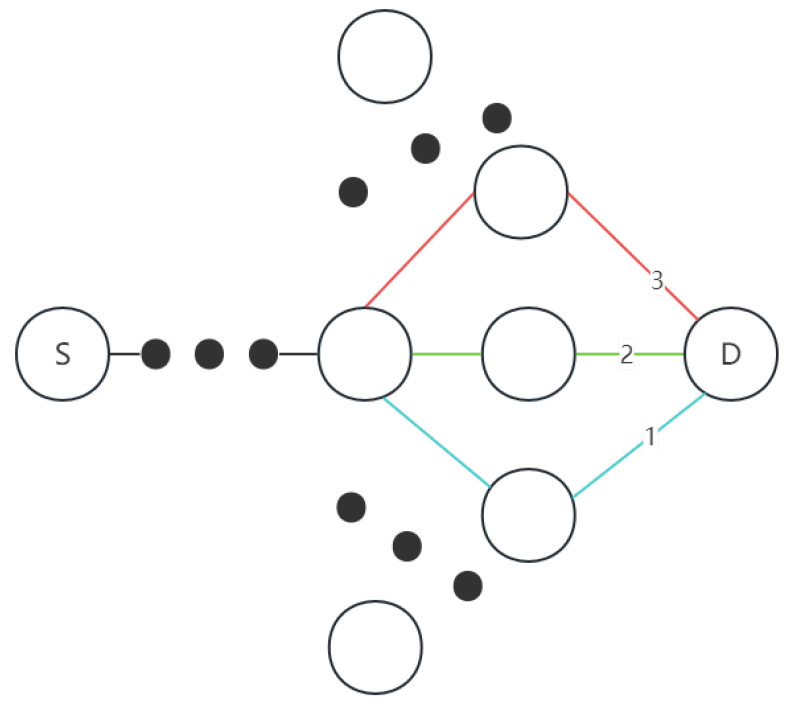
Similar paths between source and destination node.

In AODV, the route request (RREQ) is broadcast to neighbor nodes, which means that every neighbor node receives the RREQ and forwards it further. When the node density is high, the frequent broadcasting of RREQ messages results in excessive network overhead. To address this issue, ND-AODV takes advantage of the observation that in high-density environments, the performance difference between the best route and alternative routes is not significantly large. Therefore, it is possible to sacrifice the exhaustive search for the absolute best route to reduce the overall network overhead. In this way, ND-AODV lowers the cost associated with finding the optimal route and achieves improved performance in dense node environments. In Figure 3, routes 1, 2, and 3 denote three possible paths to reach the destination node, S represents the source node and D represents the destination node. The optimal route (route 2) and alternative routes (routes 1 and 3) do not exhibit significant differences. Even if routes 1 or 3 are selected, they can still effectively complete data transmission.

### 3.2. ND-AODV

In the AODV route discovery process, when a node receives a route request (RREQ) and determines that it is not the destination node and does not have a route to the destination in its routing table, it will add its own information and then forward the RREQ to its neighbor nodes. However, ND-AODV modifies this route discovery process. In [6], ND-AODV introduces a parameter called “*k*” to determine if a node has too many neighbor nodes, indicating whether the node is in a high-density environment. The optimal value for “*k*” was determined to be 11 in the experiments presented in [6]. When the number of neighbor nodes exceeds the value of “*k*” there is a probability that the node will not forward the RREQ. To calculate the probability $p_{i}$ of the forwarding RREQ of the *i* node:

(1) $$p_{i}=min\left(1,\frac{k}{Nb_{i}}\right)$$

where $Nb_{i}$ is the number of neighbor nodes.

**Figure 4:**
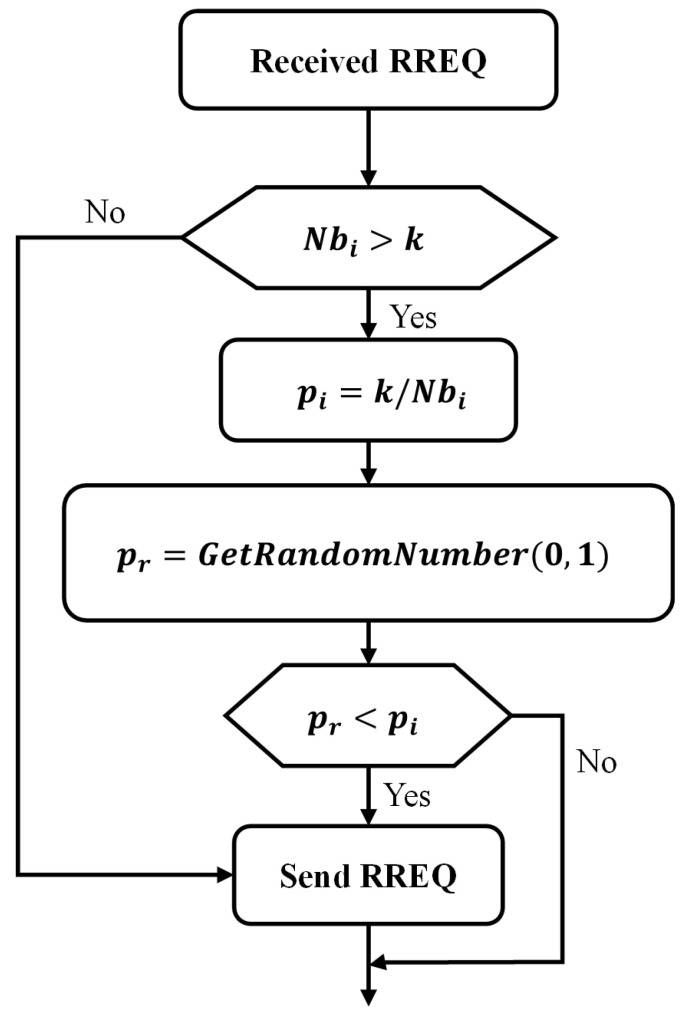
Flowchart depicting the route discovery process in the ND-AODV protocol.

In the ND-AODV route discovery process, when a node receives an RREQ and completes the destination node information check, it will then further assess whether the number of its neighbor nodes exceeds the “*k*” value. If the number is not greater than “*k*”, the node will forward the RREQ. However, if the number of neighbor nodes is greater than *k*, the node will calculate a new forwarding probability $p_{i}$ . Subsequently, it randomly generates a value $p_{r}$ within the range of (0,1) . If $p_{r}$ is less than $p_{i}$ , the node will forward the RREQ; otherwise, it will discard the RREQ and not forward it, as shown in Figure 4.

### 3.3. The Issues with ND-AODV

ND-AODV still has limitations because it lacks information about the location of neighbor nodes, which can result in two types of network wastage.

Two types of nodes are defined here, the receiving node as receiver and the sending node as sender.

**Figure 5:**
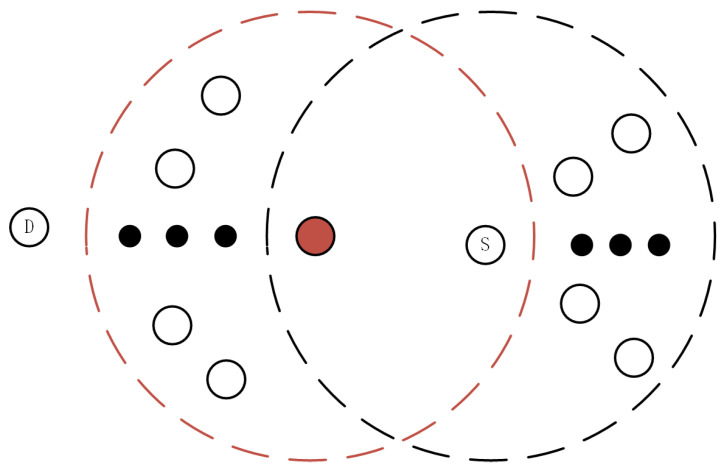
Illustration of isolated receiver in ad hoc networks, isolated node.

Scenario 1: In certain circumstances, when a sender is surrounded by an isolated receiver, meaning there are fewer common neighbor nodes between the sender and receiver, and the number of neighbor nodes around the receiver exceeds the threshold “*k*”, and this isolated node is associated with the routing to the destination node, it may have an adverse effect on the discovery of the destination node, leading to an increase in network overhead, as illustrated in Figure 5. In this context, the red node represents the receiver, S represents the sender, the dashed lines represent the range of node transmission, and D represents the destination node. When the sender transmits an RREQ to the receiver, if there is an isolated receiver, and the number of neighbor nodes around this receiver exceeds the threshold “*k*”, according to the conditions of the ND-AODV protocol, as shown by the red receiver in Figure 5, there is a certain probability of not forwarding the RREQ, making it challenging to discover the destination node D, and consequently further increasing network overhead. Therefore, this node is referred to as an isolated node.

**Figure 6:**
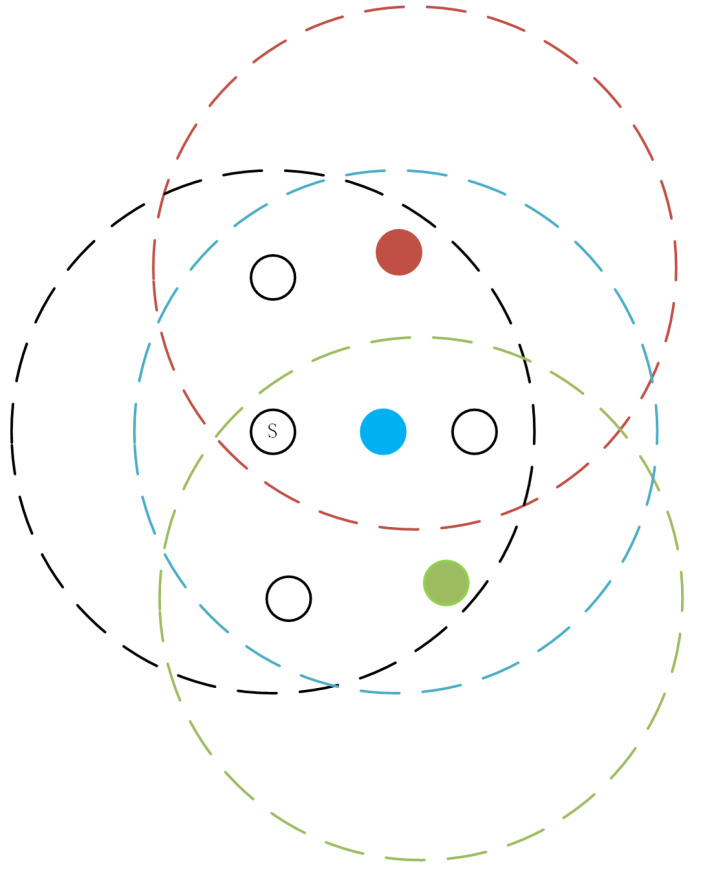
Illustration of over-dense receiver in ad hoc networks, over-dense node.

Scenario 2: In another scenario, there is an over-density of nodes in the vicinity, as shown in Figure 6, where the red, blue, and green nodes are all neighbor nodes of the sender S. If there are excessive receivers around the sender S (meaning there are many common neighboring nodes), based on the observation from the figure, the over-dense blue receiver and the sender S share many common neighbor nodes. Leveraging the characteristic of the ND-AODV protocol, which sacrifices the best route, the over-dense blue receiver can choose not to forward the RREQ control information, as indicated by the blue node in Figure 6. Specifically, when the sender transmits an RREQ to the receiver, if there is an over-dense receiver, meaning there are more neighbor nodes around both the sender and receiver than the threshold “*k*”, these over-dense receivers may still forward the RREQ with a certain probability. This can lead to the redundant propagation of control information, resulting in the wastage of network resources. Therefore, this node is referred to as an over-dense node.

## 4. Proposed New Routing Method

### CND-AODV

**Figure 7:**
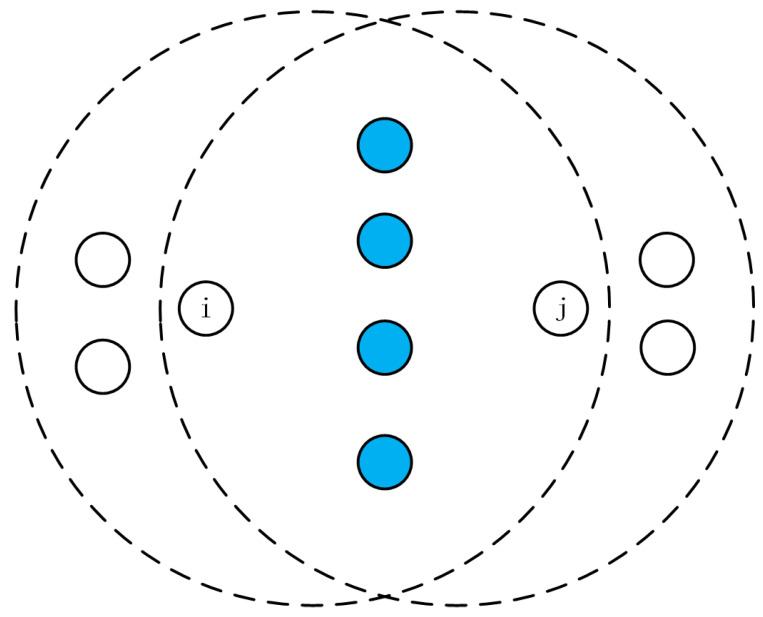
Illustration of the number of common neighbor nodes.

Based on the analysis from the previous sections, it is evident that both isolated nodes and over-dense nodes are determined based on the common neighbor nodes between the receiver and sender. If it is judged as an isolated node, the RREQ is forwarded with a probability of 100%. If it is judged as an over-dense node, the RREQ is discarded directly. Motivated by that, in this subsection, we propose a solution called CND-AODV (common neighborhood density AODV) to address those issues, which will be detailed in the followings. Figure 7 illustrates the basic idea of the common neighbor nodes, where *N* represents the number of common neighbor nodes between two adjacent nodes *i* and *j*, and the number of blue nodes in the figure is shown as $N_{ij}$ . In the process of sending a route request at a node, the basic parameters required by CND-AODV can be obtained by carrying its corresponding routing table and neighbor table.

Therefore, the number of common neighbor nodes between the sender *j* and the receiver *i* is denoted as $N_{ij}$ .

(2) $$N_{ij}=S_{i}\cap S_{j}$$

where $S_{i}$ and $S_{j}$ are the collection of neighbor nodes of receiver *i* and sender *j*.

Then, for the judgment of isolated nodes and over-dense nodes, simply speaking, an isolated node means that the $N_{ij}$ value is small, and an over-dense node means that the $N_{ij}$ value is large. So, it is necessary to analyze how to define the smaller and larger values.

It is obvious that when the sender *j* forwards control information, each of its neighbor nodes will receive it. Each receiver will have the number of common neighbor nodes $N_{ij}$ with the sender. Thus, the mean $\bar{N_{j}}$ of the number of common neighbor nodes between sender *j* and its neighbor nodes is suitable to represent the density of the neighbor nodes, i.e.,

(3) $$\bar{N_{j}}=\frac{\sum_{i=1}^{n}N_{ij}}{Nb_{j}}$$

where $Nb_{j}$ is the number of neighbor nodes of the sender *j*. Therefore, it can be defined that when the $N_{ij}$ of receiver *i* and sender *j* far exceeds the $\bar{N_{j}}$ of the sender *j*, it can be defined as an over-dense node; if it is much smaller than $\bar{N_{j}}$ , it can be defined as an isolated node.

Next, we will present a discriminative example of common neighbor nodes to illustrate the proposed method. The number of nodes $N_{ij}$ in common between the sender *j* and its neighbors will be counted, and the number of neighbors of the sender exceeds ”*k*”.

**Figure 8:**
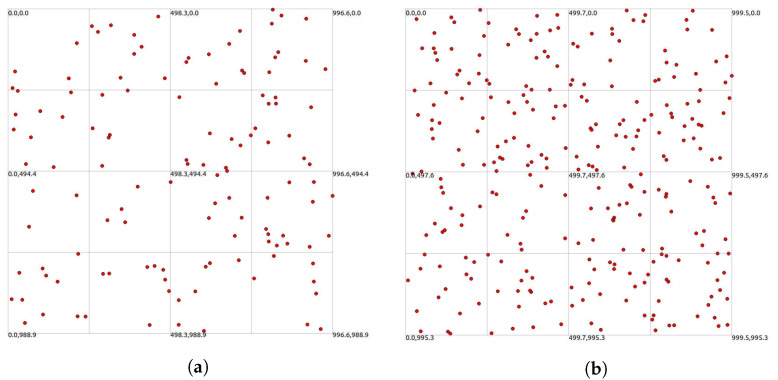
The initial simulation plots with 125 (**a**) and 275 (**b**) nodes under random distribution.

**Figure 9:**
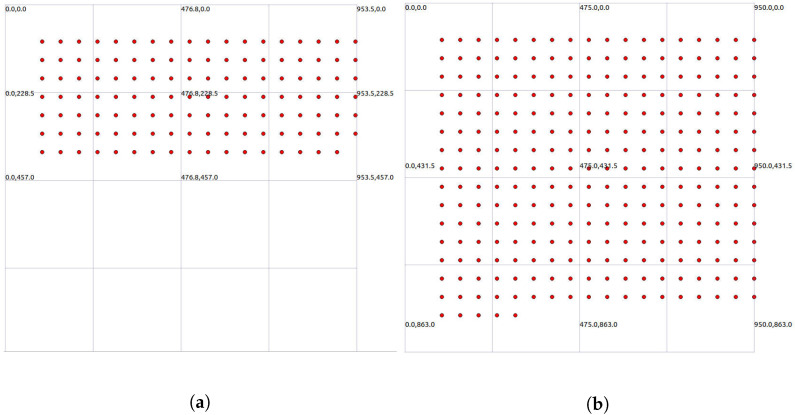
The initial simulation plots with 125 (**a**) and 275 (**b**) nodes under uniform distribution.

In the simulation experiment, with an area of 1000×1000 m^2^, two different methods were employed for node generation: random distribution and uniform distribution. These experiments involved two scenarios with different node counts, specifically 125 (it was observed that when the number of nodes is less than or equal to 75, it is difficult to form cases where the number of neighbor nodes exceeds ”*k*”) and 275. For random distribution, node positions were non-fixed, while uniform distribution represents an extreme and special case of random distribution. Additionally, each node was assigned a random motion direction upon appearance, with their speeds randomly set between 0 and 5 m/s. As shown in Figure 8 and Figure 9, the initial simulation plots for 125 and 275 nodes under random and uniform distribution illustrate the spatial distribution of nodes in the simulation environment. The left graph represents 125 nodes, while the right graph represents 275 simulated nodes. Since uniform distribution is an extreme and special case of random distribution, it is primarily considered to analyze whether there are any impacts on network performance in extreme scenarios. Therefore, the simulation experiments in Section 5 will focus on analyzing the influence of CND-AODV on network performance after nodes are generated with uniform distribution. Subsequent analyses will be based on scenarios where nodes are generated through random distribution.

**Figure 10:**
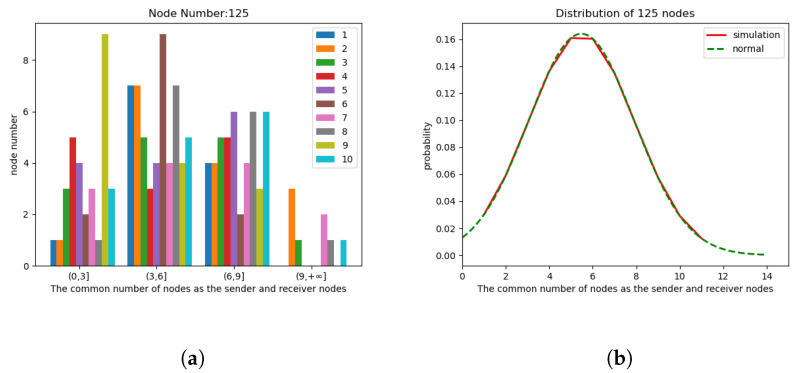
The count distribution diagram (**a**) and probability distribution diagram (**b**) of the number of common neighbor nodes of 125 nodes.

**Figure 11:**
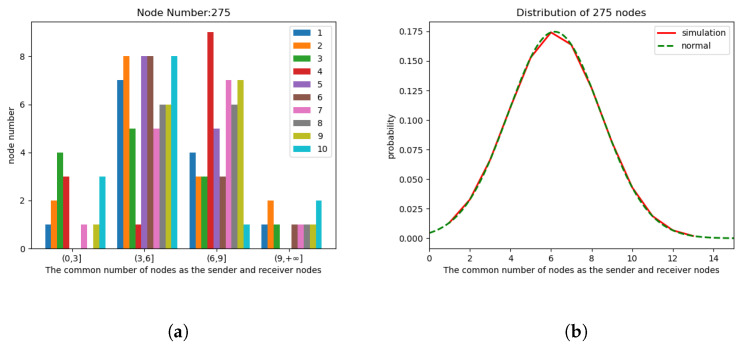
The count distribution diagram (**a**) and probability distribution diagram (**b**) of the number of common neighbor nodes of 275 nodes.

Randomly selecting 10 groups of sender *j*, we can analyze the number of common neighbor nodes $N_{ij}$ between each sender and its neighbor nodes. The data based on the number of common neighbor nodes are grouped and the statistical analysis uses the following intervals: (0,3] , (3,6] , (6,9] , and (9,∞] . The count distribution and probability distribution plots for 125 nodes are shown in Figure 10. The left Figure 10a illustrates the distribution of $N_{ij}$ values, while the right Figure 10b illustrates the probability distribution. In the probability distribution plot, “normal” indicates a standard Gaussian Distribution with the same mean and standard deviation. Similarly, Figure 11 represents the distribution and probability distribution plots for the scenario with 275 nodes.

After obtaining the simulation results and the probability distribution plot, a quantitative analysis was performed to compare the simulated distribution with the standard Gaussian distribution. This comparison was performed using the Kolmogorov–Smirnov (KS) test [21,22].

(4) $$D=\max_{1\leq x\leq 14}(|F\left(x_{i}\right)-S\left(x_{i-1}\right)|,|F\left(x_{i}\right)-S\left(x_{i}\right)\left|\right)$$

where $F(x_{i})$ is the theoretical cumulative probability distribution function, $S(x_{i})$ is the empirical or observed cumulative probability distribution function for the variable $x_{i}$ , which in this case is the $N_{ij}$ values, and *D* is the statistical measure for the maximum difference between two different cumulative distribution functions. Based on the obtained results for the two scenarios with 125 and 275 nodes, the maximum *D* values were found to be 0.114 and 0.111, respectively. The critical value for the KS test depends on the sample size (*n*) and the desired significance level ( α , its value is 0.5). By using the threshold expression from reference [22]:

(5) $$\hat{D}=\frac{1.36}{\sqrt{n}}$$

where $\hat{D}$ is the threshold for the maximum difference statistic of *D*, a constant used to determine whether a distribution is normal, n is the the sample size of 125 and 275, and considering the critical significance level ( α ) typically set to 0.05, we can calculate the critical values $\hat{D}$ as 0.119 and 0.116, respectively. As the obtained *D* values of 0.114 (for 125 nodes) and 0.111 (for 275 nodes) are less than their respective critical values ( $\hat{D}=0.119$ and $\hat{D}=0.116$ ), we can conclude that, with a high probability, the observed cumulative probability distribution function *S*(*x*) follows the trend of a standard Gaussian distribution for the common neighbor node count in the range of 1 to 14. This can also be referred to as a truncated Gaussian, observed in scenarios with node counts of 125 and 275. Therefore, we can infer that this trend is likely to hold for node quantities within the range between 125 and 275 as well.

Correctly, based on the demonstrated trend of $N_{ij}$ following a truncated Gaussian, the focus can be on identifying nodes that deviate significantly from the mean ( $\bar{N_{j}}$ ). In the experiment, z-scores [23] can be utilized as a statistical measure to assess the relative position or deviation of a data point within a dataset. Specifically, the z-score represents the number of standard deviations between a data point and the mean. By using z-scores, one can determine the position of a data point within the entire dataset and assess its deviation from the mean:

(6) $$z=\frac{X-\mu }{\sigma }$$

where *z* is the z-score, *X* is the value of the data point, μ is the mean of the dataset, and σ is the standard deviation of the dataset.

In order to better evaluate the degree of deviation, it is necessary to calculate the standard deviation $▵c_{j}$ of the number of common neighbor nodes $N_{ij}$ between sender *j* and its neighbor nodes, i.e.,

(7) $$▵c_{j}=\sqrt{\frac{\sum_{i=1}^{n}{\left(N_{ij}-\bar{N_{j}}\right)}^{2}}{Nb_{j}}}$$

According to the z-scores method, the specific expression for calculating z-scores in the data is as follows:

(8) $$z=\frac{N_{ij}-\bar{N_{j}}}{▵c_{j}}$$

Then, the relative z-scores of nodes in the data can be obtained, where positive values indicate being above the mean, with larger values indicating a greater deviation from the mean. On the other hand, negative values indicate being below the mean, with smaller values indicating a greater deviation from the mean. Then, the z-scores method involves different confidence intervals with varying proportions, as shown in Table 2.

Thus, nodes that fall outside the confidence intervals can be considered to have significant deviations and can be defined as isolated nodes or over-dense nodes.

Isolated node: When the z-score generated from the number of common neighbor nodes $N_{ij}$ between the receiver *i* and the sender *j* is less than the negative z-threshold, the receiver *i* is considered an isolated node of the sender *j*. In the concrete route discovery process, when the receiver *i* receives the RREQ and confirms that it does not meet the destination node conditions, and the number of its neighbor nodes exceeds the threshold *k*, it further checks whether the receiver *i* is an isolated node of the sender *j*. If it is determined to be an isolated node, it will forward the RREQ with a probability of 100% , otherwise, it will perform a probability calculation to decide whether to forward the RREQ.

Over-dense node: When the z-score generated from the number of common neighbor nodes $N_{ij}$ between the receiver *i* and the sender *j* is greater than the positive z-threshold, the receiver *i* is considered an over-dense node of the sender *j*. In the concrete route discovery process, when the receiver *i* receives the RREQ and confirms that it does not meet the destination node conditions, and the number of its neighbor nodes exceeds the threshold ”*k*”, it further checks whether the receiver *i* is an over-dense node of the sender *j*. If it is determined to be an over-dense node, the RREQ is directly discarded; otherwise, it will perform a probability calculation to decide whether to forward the RREQ.

**Figure 12:**
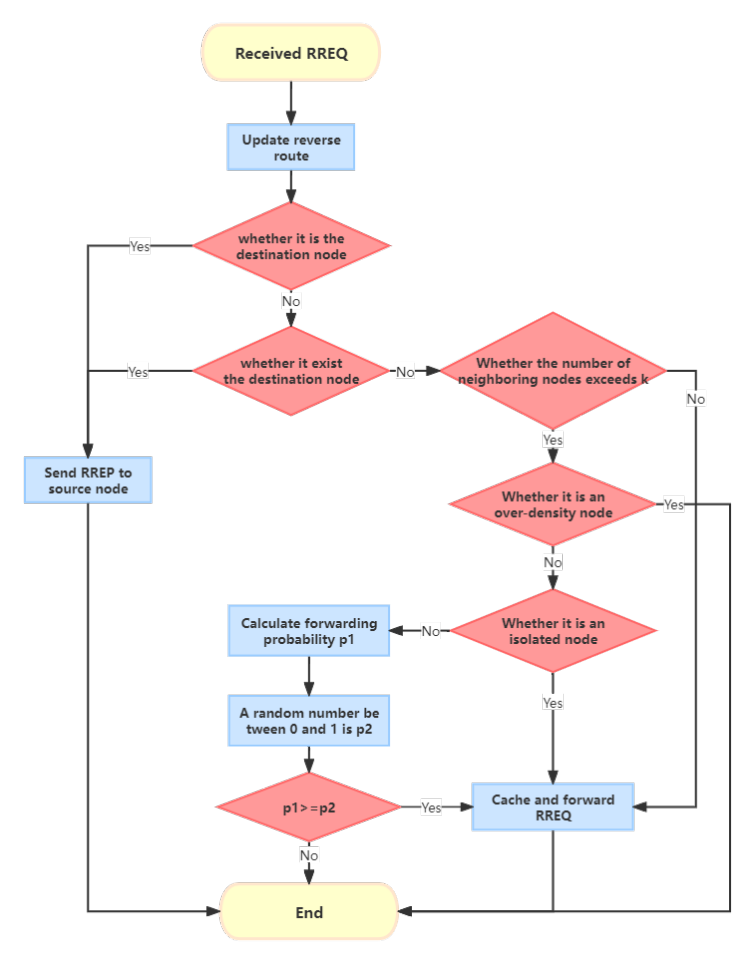
Flowchart depicting the route discovery process in the proposed CND-AODV protocol.

Based on the above analysis, we will obtain a new route discovery method, and its flowchart can be expressed in Figure 12.

## 5. Simulations and Results

### 5.1. Simulations

#### 5.1.1. Simulation Environment

We mainly simulated three protocols: AODV, ND-AODV, and CND-AODV using the NS3.35 simulation platform. NS3 [24] is a powerful network simulation tool used for modeling and analyzing various network scenarios, including wired networks, wireless networks, mobile networks, satellite networks, and internet protocols, among others. NS3 is known for its stability, robust functionality, easy extensibility, and being a free network simulation software. NS2 [25] is a widely used discrete-event network simulator, primarily used for studying and evaluating network communication algorithms. However, NS2 has not been updated in recent years, and its user base has declined. NS3, on the other hand, offers better performance, greater flexibility, superior documentation, and strong community support, making it a more suitable choice for meeting users’ simulation needs.

In this experiment, we used a simulation time of 110 s, a simulation area of 1000×1000 m^2^, and a TwoRayGroundPropagationLossModel as the propagation loss model. The packet size for transmission was set to 512b, the transmission rate was 2 Mbps, and the transport layer protocol was UDP. The transmission model used was CBR (constant bit rate), and the node mobility model was set as RandomWaypointMobilityModel, see Table 3.

The experiments cover two different environments. Environment 1: Nodes are randomly distributed, and various threshold values (*z*) are introduced for comparison. Simulations are conducted by altering the number of nodes to obtain the optimal threshold parameter (*z*). Environment 2: Scenario 1—varying node count; scenario 2—nodes are uniformly distributed to assess CND-AODV performance in extreme conditions; scenario 3—changing node movement speeds. In each scenario, the performance differences among the different protocols are compared.

#### 5.1.2. Metrics

We will evaluate the performance of the proposed algorithm based on the network’s throughput, delay, and packet delivery ratio [26,27]. These metrics will help us assess the effectiveness and efficiency of the algorithm in comparison to other approaches.

Throughput: The total number of received data packets in a unit of time.

(9) $$\mathrm{Throughput}=\sum \frac{\mathrm{Receive}\;\mathrm{bytes}\times 8}{\mathrm{Simulation}\;\mathrm{Time}\times 1024}$$

Delay: Also known as average delay, it represents the time taken from the source node sending a data packet to the destination node receiving it.

(10) $$\mathrm{Average}\;\mathrm{Delay}=\sum \frac{{\mathrm{Time}}_{\mathrm{packetReceive}}-{\mathrm{Time}}_{\mathrm{packetSent}}}{\mathrm{Total}\;\mathrm{Number}\;\mathrm{of}\;\mathrm{Packet}\;\mathrm{Received}}$$

Packet delivery ratio: The ratio of the number of data packets received at the destination node to the number of data packets sent by the source node.

(11) $$\mathrm{Packet}\;\mathrm{Delivery}\;\mathrm{Ratio}=\frac{\sum \mathrm{Packet}\;\mathrm{Received}}{\sum \mathrm{Packet}\;\mathrm{Sent}}\times 100\%$$

### 5.2. Result

#### 5.2.1. Environment 1

**Figure 13:**
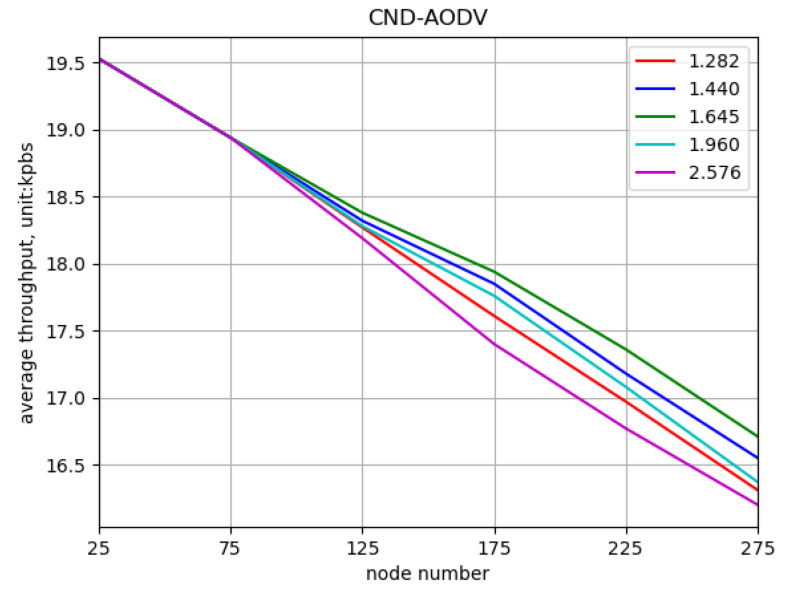
Throughput performance of CND-AODV with different threshold values (*z*).

**Figure 14:**
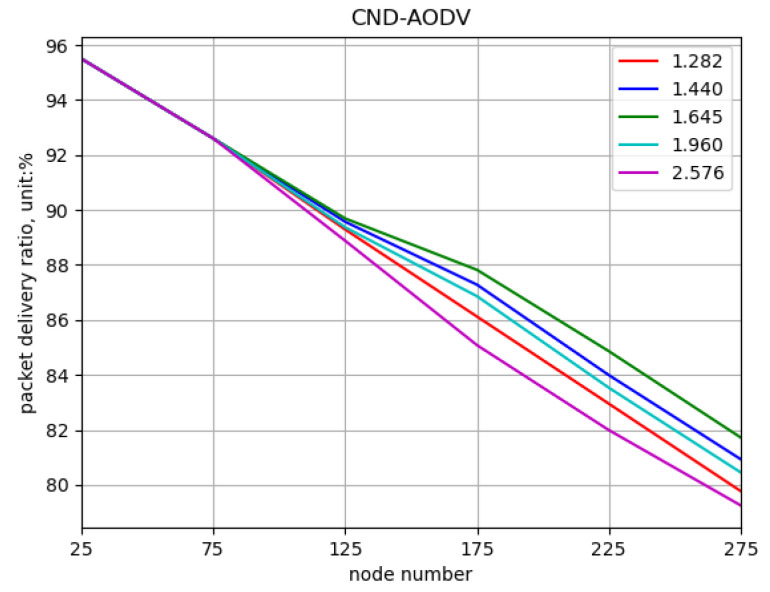
Packet delivery ratio performance of CND-AODV with different threshold values (*z*).

**Figure 15:**
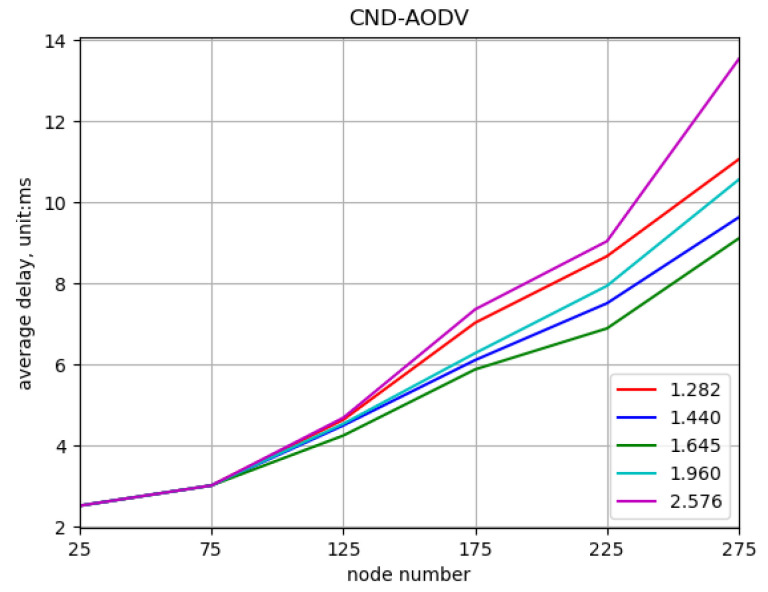
Delay performance of CND-AODV with different threshold values (*z*).

Comparing different values of the threshold parameter “*z*” to obtain the optimal value. In this simulation, the network is composed of nodes with numbers 25, 75, …, 275, and the nodes’ movement speeds vary randomly between 0 and 5 m/s at different time intervals. The threshold values for “*z*” are set to 1.282, 1.440, 1.645, 1.960, and 2.576, where higher values indicate fewer nodes with significant deviations. The performance of the CND-AODV protocol in the mobile ad hoc network is evaluated for different “*z*” values, as shown in Figure 13, Figure 14 and Figure 15. From Figure 13, Figure 14 and Figure 15, it can be observed that regardless of the “*z*” value, both the throughput and packet delivery ratio exhibit a decreasing trend, and the delay demonstrates an increasing trend. However, when the threshold value “*z*” is set to 1.645, corresponding to the 90% confidence interval, there is a slight improvement in both throughput and packet delivery ratio compared to other values. Additionally, the increase in delay is reduced, resulting in a minor improvement as well.

#### 5.2.2. Environment 2

According to the previous experimental results, it was demonstrated that CND-AODV performs best when the threshold value “*z*” is set to 1.645. Therefore, in this section of the experiment, the CND-AODV protocol will have its “*z*” threshold parameter set to 1.645.

Scenario 1: Scenario with varying numbers of nodes, the experiment consists of varying the number of nodes from 25 to 275, with node movement speeds randomly set between 0 and 5 m/s, simulating normal walking and cycling scenarios for data transmission.

**Figure 16:**
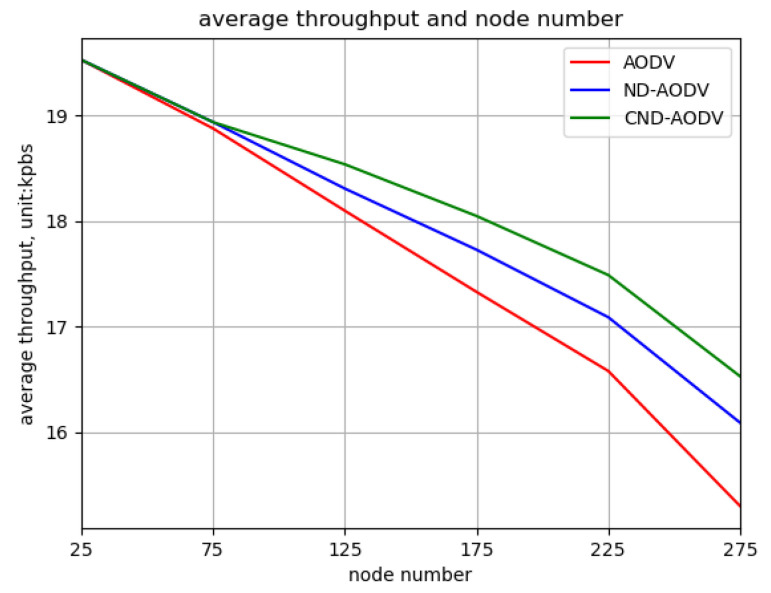
Relationship between throughput and number of nodes for different protocols under random distribution.

**Figure 17:**
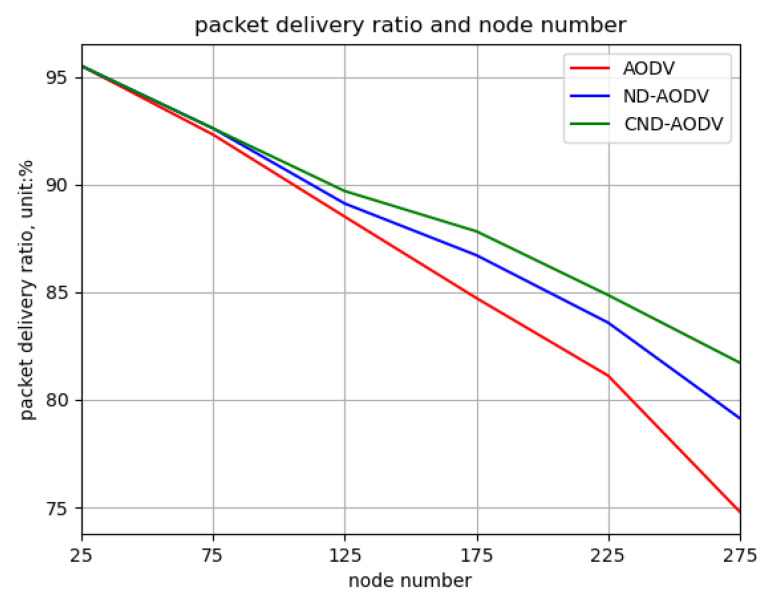
Relationship between packet delivery ratio and number of nodes for different protocols under random distribution.

**Figure 18:**
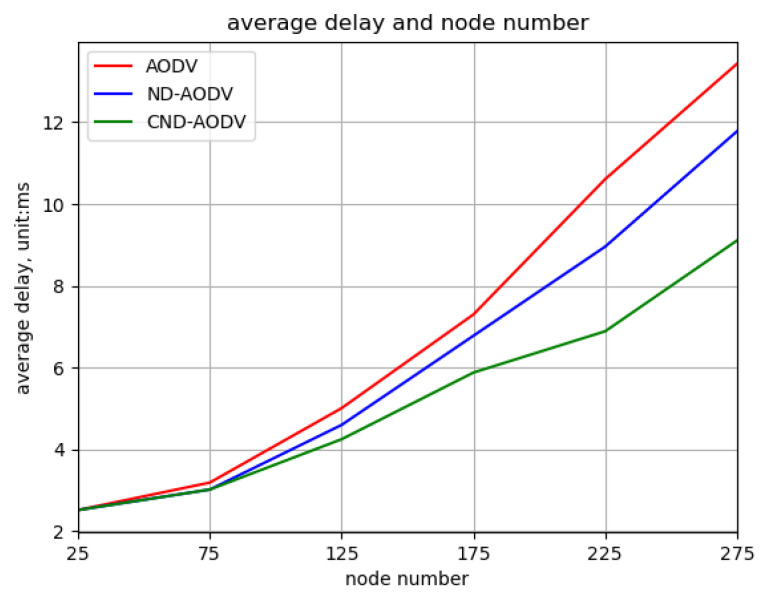
Relationship between delay and number of nodes for different protocols under random distribution.

As shown in Figure 16 and Figure 17, it can be observed that as the number of nodes increases from 25 to 275, the throughput and packet delivery ratio of three routing methods, including AODV, ND-AODV, and the proposed CND-AODV, all exhibit a gradual decrease. However, CND-AODV shows the smallest decline in performance. As depicted in Figure 18, regarding the delay, with the continuous increase in the number of nodes, the network delay for three protocols demonstrates an upward trend. Nonetheless, it is evident that CND-AODV significantly outperforms AODV and ND-AODV in terms of transmission delay. In summary, in scenarios with a larger number of nodes, CND-AODV exhibits notable improvements in both throughput and network delay.

Scenario 2: This involves simulation experiments with different node counts using a z-threshold of 1.645. Under uniform distribution conditions, the network performance of AODV, ND-AODV, and CND-AODV is compared. The specific distribution is illustrated in Figure 9, and the performance of CND-AODV under uniform distribution is analyzed by comparing the throughput, packet delivery rate, and delay.

**Figure 19:**
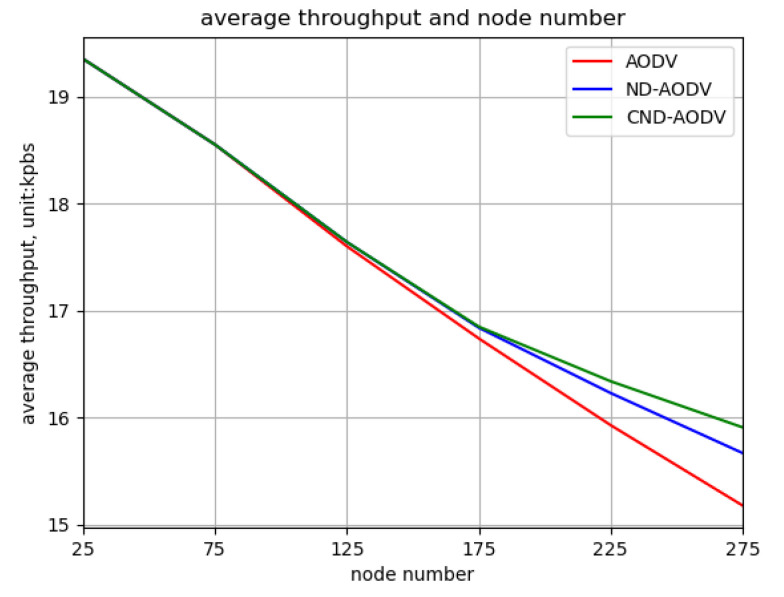
Relationship between throughput and number of nodes for different protocols under uniform distribution.

**Figure 20:**
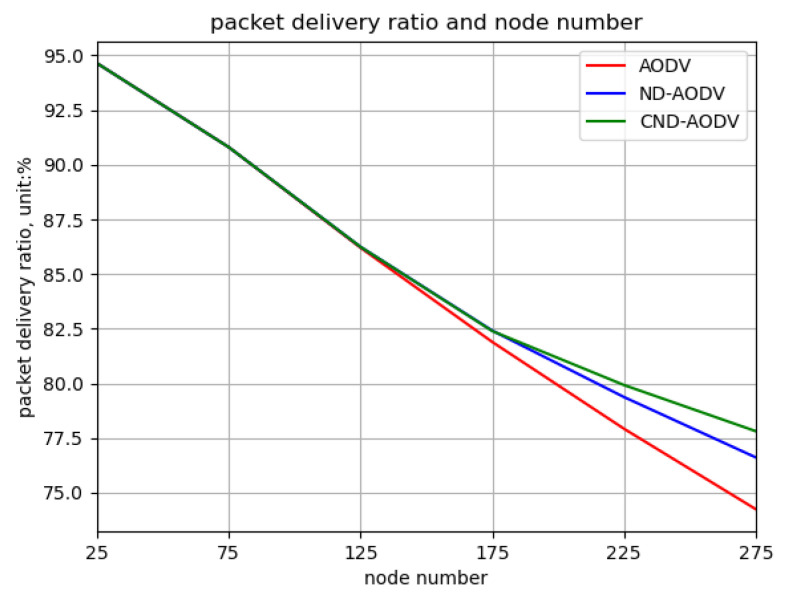
Relationship between packet delivery ratio and number of nodes for different protocols under uniform distribution.

**Figure 21:**
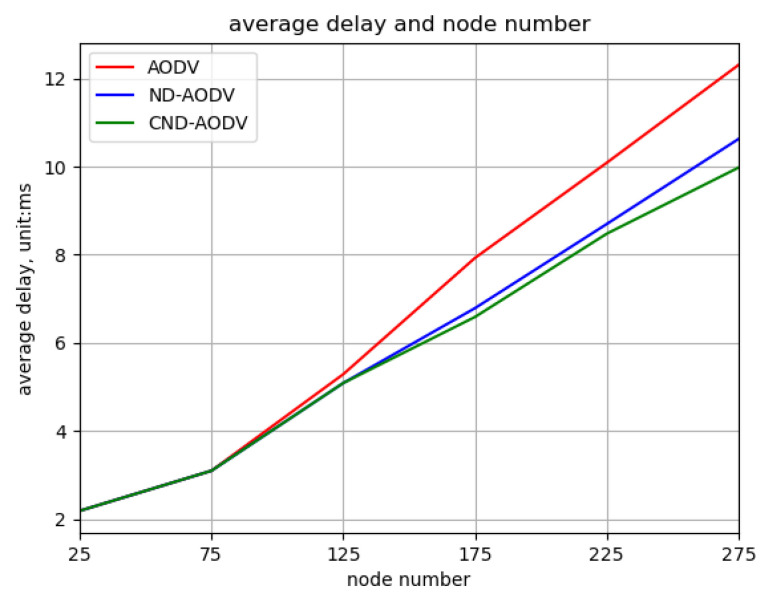
Relationship between delay and number of nodes for different protocols under uniform distribution.

In the scenario of uniform node distribution, as the node count increases from 25 to 275, it can be observed from Figure 19 and Figure 20 that both the throughput and packet delivery rate exhibit a decreasing trend. However, the performance of ND-AODV and CND-AODV surpasses that of AODV, and this advantage becomes more pronounced with the increase in node count, with CND-AODV slightly outperforming ND-AODV. As depicted in Figure 21, the delay increases with the growing number of nodes for all three protocols, but ND-AODV and CND-AODV show a slower growth rate, with CND-AODV slightly outperforming ND-AODV. Therefore, under uniform distribution conditions, it can be concluded that the performance of CND-AODV is slightly superior to ND-AODV and significantly better than AODV.

Scenario 3: Scenario with varying speeds of nodes; in this experiment, the node movement speeds are randomly set to 3 m/s, 25 m/s, and 50 m/s, corresponding to pedestrian, vehicular, and high-speed highway vehicular scenarios, respectively. The number of nodes is fixed at 200 for data transmission.

**Figure 22:**
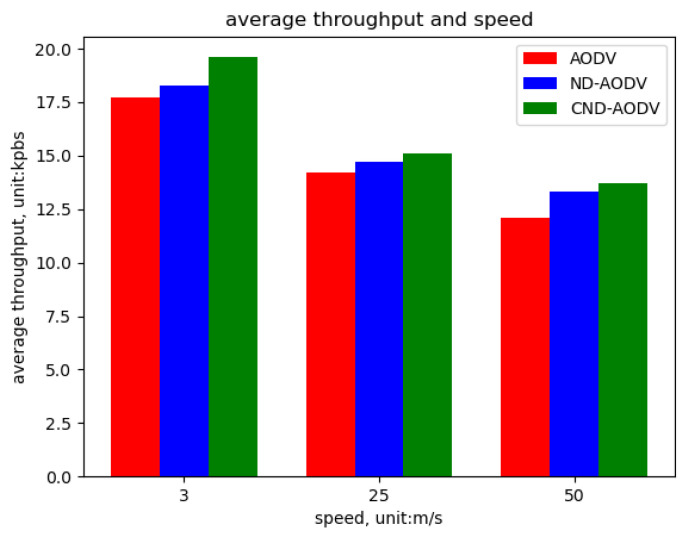
Throughput performance with respect to the speed of nodes for different protocols.

**Figure 23:**
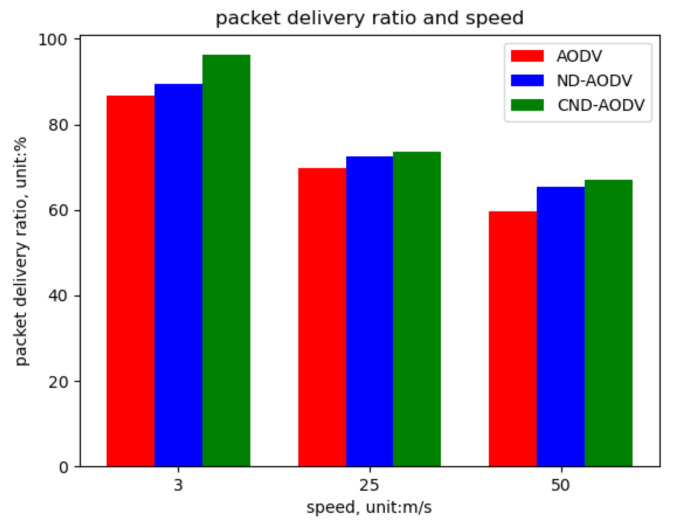
Packet delivery ratio performance with respect to the speed of nodes for different protocols.

**Figure 24:**
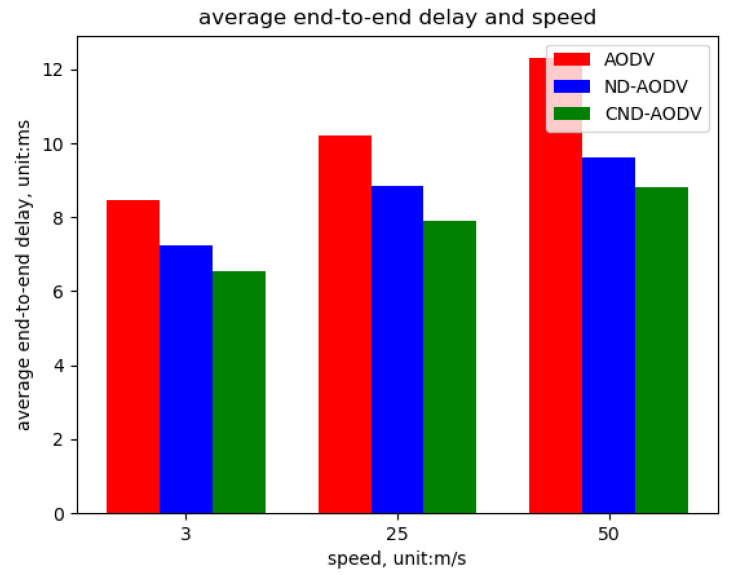
Delay performance with respect to the speed of nodes for different protocols.

As shown in Figure 22, Figure 23 and Figure 24, we can observe that our proposed algorithm CND-AODV exhibits a gradual decline in throughput and packet delivery ratio as the node movement speed increases. However, it still slightly outperforms ND-AODV and AODV. As the node movement speed increases, the average delay of CND-AODV also shows an increasing trend, but it remains lower than those of AODV and ND-AODV. Therefore, with the increase in node movement speed, CND-AODV shows some improvement in network performance.

As depicted in Figure 22 and Figure 23, it can be observed that as the speed of nodes increases, the throughput and packet delivery ratio of the three routing methods, including AODV, ND-AODV, and the proposed CND-AODV, all exhibit a decline. However, CND-AODV shows the smallest decline in performance. As shown in Figure 24, regarding the delay, with the continuous increase in the speed of nodes, the network delay for all three methods demonstrates an upward trend. Nevertheless, it is evident that CND-AODV outperforms AODV and ND-AODV in terms of transmission delay during the process of increasing delays. Therefore, in scenarios with higher node mobility speeds, CND-AODV shows a certain level of improvement in network performance.

## 6. Conclusions

In this study, an improved routing protocol called CND-AODV was proposed to address the issue of network storms caused by the broadcast of routing control messages in mobile ad hoc networks. This protocol takes into account both the node density and the number of common neighbor nodes to determine if a node is isolated or over-dense, and then utilizes probabilistic forwarding to reduce network overhead. Through experimental simulations, it was found that under appropriate threshold settings (e.g., *z* = 1.645), CND-AODV outperforms traditional AODV and enhanced ND-AODV in terms of throughput, packet delivery ratio, and delay. Simulations in various scenarios, involving changes in node count, random distribution of nodes, and extremely uniform distribution, indicate that CND-AODV enhances network performance. This improvement is more significant when nodes are randomly distributed. Additionally, CND-AODV exhibits improved network performance in scenarios with varying node mobility rates. Thus, CND-AODV proves to be an effective routing protocol, successfully mitigating network storms caused by routing control information broadcasts in mobile ad hoc networks and exhibiting superior performance across diverse scenarios. This study introduces a novel approach to optimizing routing in mobile ad hoc networks, offering valuable insights for future research and practical applications in related fields.

Note that in CND-AODV, the determination of isolated and over-dense nodes relies on the number of common neighbor nodes and the z-scores method. It is yet to be confirmed whether this determination method is applicable to all network scenarios, and further validation is needed in this regard. 

## Figures and Tables

**Table 1:** General variables and parameters.

$Nb_{i}$	Number of neighbor nodes of node *i*
$p_{i}$	Probability $p_{i}$ of the forwarding RREQ of node *i*
$p_{r}$	Randomly generated probability, used to compare with $p_{i}$
*k*	Constant threshold, for congestion detection
$N_{ij}$	Number of common neighbor nodes between node *j* and node *i*
$\bar{N_{j}}$	Mean of the number of common neighbor nodes between sender *j* and its neighbor nodes
$S_{i}$	The set of neighbor nodes of node *i*
$F(x_{i})$	Theoretical cumulative probability distribution function
$S(x_{i})$	Empirical or observed cumulative probability distribution function
*D*	Statistical measure for the maximum difference between two different cumulative distribution functions
$\hat{D}$	The threshold for the maximum difference statistic of D
*z*	The *z*-score, utilized as a statistical measure to assess the relative position or deviation of a data point within a dataset
*X*	The value of the data point, used to calculate *z*
μ	Mean of the dataset, used to calculate *z*
σ	Standard deviation of the dataset, used to calculate *z*
$▵c_{j}$	Standard deviation of the number of common neighbor nodes $N_{ij}$ between sender *j* and its neighbor nodes

**Table 2:** The different confidence intervals using different thresholds of *z*.

Threshold *z*	Confidence Interval
−1.282≤z≤1.282	80%
−1.440≤z≤1.440	85%
−1.645≤z≤1.645	90%
−1.960≤z≤1.960	95%
−2.576≤z≤2.576	99%

**Table 3:** Simulation parameters of NS3.

Parameter	Value
Simulation Platform	NS3.35
Simulation Time	110 s
Simulation Protocol	AODV, ND-AODV, and CND-AODV
Number of Nodes	25, 75, 125, 175, 200, 225, 275
Active Nodes	10
MAC Protocol	IEEE 802.11p
Simulation Area	1000×1000 m^2^
Propagation Loss Model	TwoRayGroundPropagationLossModel
Speed of nodes	3, 5, 25, and 50 m/s
Packet Size	512b
Mobility Model	RandomWaypointMobilityModel
Transport Layer Protocol	UDP
Transmission Model	CBR (constant bit rate)
Transmission Rate	2 Mbps

## Data Availability

Data are contained within the article.
